# Anti-Acne Action of Peptides Isolated from Burdock Root—Preliminary Studies and Pilot Testing

**DOI:** 10.3390/molecules25092027

**Published:** 2020-04-27

**Authors:** Malgorzata Miazga-Karska, Katarzyna Michalak, Grazyna Ginalska

**Affiliations:** 1 Department of Biochemistry and Biotechnology, Medical University of Lublin, Chodzki 1, 20-832 Lublin, Poland; g.ginalska@umlub.pl; 2 Department of Epizootiology, Clinic of Infectious Diseases, University of Life Sciences, Gleboka 30, 20-612 Lublin, Poland; kat.michalak86@gmail.com

**Keywords:** Antibacterial peptides, acne skin, polysaccharide dressing, *Arctium lappa L.*

## Abstract

This work aimed to study the anti-bacterial, anti-biofilm and anti-oxidant potential effects of low molecular weight (LMW) peptides (Br-p) isolated from burdock (*Arctium lappa L.*) roots. We conducted a preliminary study to exclude or confirm the antibiotic activity of the LMW peptides fraction of this plant. Br-p were isolated using gel filtration and a 10 kDa cut-off membrane. The obtained peptides were identified by MALDI TOF/TOF. Antibacterial activity was tested against acne strains using diffusion tests, MIC and MBC. The fibroblast cytotoxicity of Br-p was tested, and the selectivity index (SI) value was determined. The fraction of 46 Br-p peptides isolated from burdock root with a molecular weight below 5000 Da and theoretic pI (isoelectric point) of 3.67–11.83 showed a narrow spectrum of activity against Gram-positive acne bacterial strains. One of the Br-p peptides assessed on MALDI RapidDeNovo was LRCDYGRFFASKSLYDPLKKRR cationic peptide. It was analogous to that contained in *A. lappa* protein, and theoretically it was matched as a peptide with antibiotic nature. Br-p did not show toxicity to fibroblasts in the tested concentration up to 10 mg/mL, obtaining CC_50_ 10 mg/mL. The SI value for the tested *Propionibacterium* strains ranged from 160 to 320. Finally, an active dressing based on chitosan/alginate/genipin was prepared using freeze-drying. The formed dressing was evaluated for its anti-acne activity. To sum up: preliminary biological studies confirmed the anti-acne properties of the isolated peptide fraction from burdock root and pointed to the possibility of using it to create an active dressing on the skin.

## 1. Introduction

The World Health Organization (WHO) warns against epidemiological threats. Today it is coronavirus, tomorrow drug-resistant bacteria can cause a large number of complications and high mortality. Irrational antibiotic therapy leads to microbial resistance, which is the ability of microorganisms (like bacteria, viruses, and some parasites) to stop antimicrobial drugs from resisting against them. As a result, standard treatments become ineffective, and infections persist and may spread to others. WHO reports that the misuse of antibiotics is putting us all at risk, and the world is heading towards a pre-penicillin era. However, if effective anti-infection medication runs out, most invasive medical procedures, from tooth extraction to complicated surgery, will again be at high risk of fatal infection [1].

*Propionibacterium* acnes is the most important skin inflammatory factor. An acne skin treatment lasting several years is a difficult and complex problem not only for dermatologists. It is also noticeable by surgeons; for patients with acne around the shoulders and the back, chest or thighs, it can cause postoperative infections and prolong healing due to the presence of *Propionibacterium* acnes. In such cases, bactericidal treatment before and after surgery is recommended. In most European countries, resistance of *Propionibacterium acne* to antibiotics mainly applies to macrolides, clindamycin and erythromycin. Before starting antiacne therapy the incidence of such resistance is 51% (Hungary) up to 94% (Spain) [2]. Therefore, it is purposeful to create new treatment strategies by searching for new drugs based on natural antibacterial peptides, which can be an alternative to classic antibiotics and prevent the emergence of resistance in bacteria.

Antimicrobial peptides (AMPs) are a part of the immune system of plants and animals. The AMP database indicates that among all 3070 registered, 75% are AMPs of animal origin (vertebrates, invertebrates), 13% plant, 8.43% bacterial, 0.47% fungal, 0.23% protozoan, and 1.58% synthesized de novo [3].

AMPs are the type of cationic peptides that include all oligo- and polypeptides (peptides that arise as a result of separation from larger proteins) that kill or inhibit microbial growth [4]. Zasloff M. was the first who observed that injured frogs secrete antibacterial substances, which he called magainins. The mechanism of their action was the attachment of magainin molecules to microorganisms and destroying of their cell membranes [5]. Membrane permeability is the main mechanism to describe the action of antimicrobial peptides [6,7,8]. Most antibacterial peptides are positively charged and have an amphiphilic character. These cationic AMPs can interact with the negatively charged bacterial cell membranes, causing changes to the electrochemical potential and the permeation of molecules. This affects the destruction of cell membranes and ultimately causes cell death [6,7,8,9]. The therapeutic use of AMP is that they are selective for the type of cell membrane. It turns out that they effectively disrupt the continuity of Prokaryotic membranes, while they do not cause damage to Eukaryotic membranes. This selectivity is possible through the differences in chemical composition between such membranes. Briefly, Eukaryotic human cell membranes have up to 50% cholesterol, while fungal membranes contain ergosterol instead, and bacteria contain no sterol at all [6]. The characteristic feature of plant peptides is the high content of cysteine residues (plant thionins and defensins) [10], which create two to six disulphide bonds that affect chemical resistance and resistance to proteolytic degradation [4]. At present, several families of antimicrobial peptides of a wide variety of plant species have been isolated from roots [4], leaves [11,12,13], flowers [14], and seeds [15,16,17,18,19].

Our work aimed to conduct preliminary studies on the isolation of low molecular weight peptides from burdock roots (*Arctium lappa L.*) and to assess their level of antibacterial activity against Gram-positive and Gram-negative bacteria. Antimicrobial activity of plant peptides has been noticed by some research teams [3,4,10,11,12,13,14,15,16,17,18,19], but there are no reports on peptides isolated from burdock. Folk and traditional medicine, especially in Asia, India and Europe, indicate the beneficial consumption of fresh, dried or marinated roots and stems of this plant. The oil from burdock root is most often used for medical purposes because it contains proteins, fat, sugar, inulin, phytosterols, essential oils, glycosides, saponins, sulfur, phosphorus and ascorbic acid [20]. The oil has antibacterial [21,22], antifungal [23] and anti-inflammatory properties [24,25], and recently was also shown to be anti-diabetic [26]. The oil preparation can be used to treat boils, acne, dandruff and other seborrheic diseases. Referring to the beneficial properties of the burdock root oil fraction, our research was directed at obtaining an aqueous peptide fraction from burdock and then assessing its biological activity.

## 2. Results

**Figure 1:**
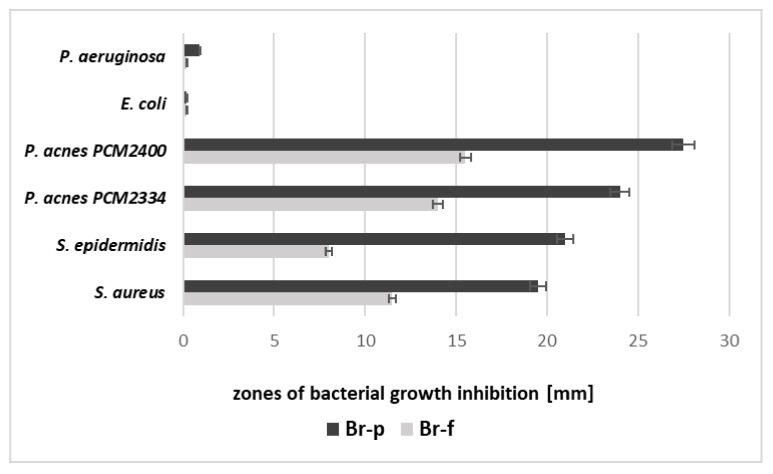
Zones of bacterial growth inhibition as antibacterial activity of Burdock samples.

The burdock roots samples, isolated using ultrasound and salting out procedure, were fractionated using a Sephadex G-50 gel filtration (Materials and Methods, Section 4.1.) The obtained fractions were evaluated for the amount of protein and antibacterial properties (the diffusion test in solid medium). The active fractions were combined and resulting in obtaining a peptide fraction named Br-f (the amount of protein for the Br-f fraction was 354 µg/mL). Br-f was initially tested for antibacterial activity (Figure 1, Table 1), and the remaining Br-f part was further ultrafiltrated in Amicon (10 kDa cut-off), followed by freeze-drying to obtain final Br-p peptide samples (the protein content was 51 µg/mL). It turns out that the antimicrobial activity increased with purification steps, during which the protein levels decreased. The data in Table 1 and Figure 1 indicate that both peptide samples of burdock root (Br-f and Br-p) did not inhibit the growth of Gram-negative bacteria. In contrast, there was a noticeable inhibitory effect on Gram-positive bacteria by both peptide samples: Br-f and the purified peptide fraction of Br-p inhibited the actions of bacterial strains, both aerobic and anaerobic. However, the purified Br-p peptide fraction worked almost twice as strong compared to Br-f. These Br-f created zones of Gram-positive bacteria growth inhibition in the range of 8.5–15 mm, while Br-p peptides showed growth inhibition of the tested strains in the range of 19.5–27.5 mm (Figure 1).

The above results were confirmed with a minimum inhibitory concentration (MIC) evaluation. Data in Table 1 indicate the most favorable, low MIC concentration values were obtained by Br-p peptide samples primarily against the acne strains *P. acnes* PCM 2334 (MIC 31.25 µg/mL) and *P. acnes* PCM 2400 (MIC 62.5 µg/mL). Higher MIC values were determined against other Gram-positive *Staphylococcus aureus* and *S. epidermidis* bacteria (MIC 250 and 500 µg/mL, respectively). However, a twice as high Br-f concentration was needed to achieve such MIC values for *S. aureus* and *S. epidermidis* and up to eight times higher to reach MICs for both *P. acnes* species. In the MIC assay, both peptide burdock samples were not active against Gram-negative strains.

Another MBC/MIC test confirmed that the bactericidal or bacteriostatic nature of burdock samples depends on the step of their purification (Table 1). The Br-p peptide fraction was bactericidal against all tested Gram-positive strains, both aerobic and anaerobic. However, Br-f fractionated using a SephadexG-50 gel filtration had only a bacteriostatic character to these Gram-positive strains. Due to the low Br-p and Br-f activity against Gram-negative strains, the MBC/MIC value could not be determined. In subsequent experiments, the Br-p peptide sample was used because of its good antibacterial activity.

**Figure 2:**
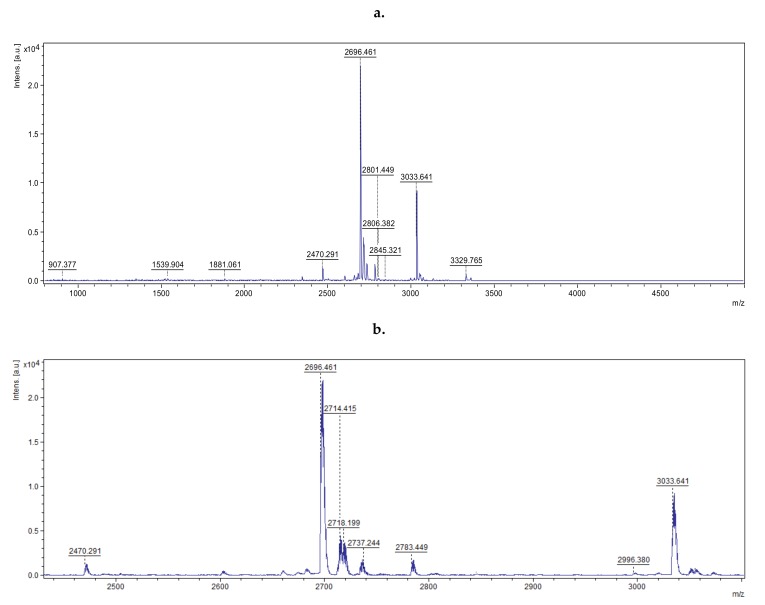
MALDI-TOF spectrum of sample peptide mixture (**a**), extension of range showing ions for MS/MS fragmentation (**b**).

In the next stage of the study, the MS spectrum of the peptide mixture was assessed by MALDI-TOF. The obtained spectrum was smoothed, and the list of peaks for the signal-to-noise ratio was digitally generated (Figure 2, Figure S1).

Obtained data confirmed that the Br-p sample consisted of 46 peptides with molecular masses lower than 5000 Da. The 10 major peaks (corresponding to peptides) with a signal-to-noise ratio greater than 20 were loaded and subjected to BioTools RapiDeNovo sequencing. The signal-to-noise parameter response to intensity of the peak corresponds to the number of certain peptides. These peaks were selected for analysis due to the supposition that they had the greatest effect on antibacterial action. The peptide sequences obtained from the Br-p burdock root sample were further analyzed using the Basic Local Alignment Search Tool (BLAST) homology base. This base allowed the peptides present in the Br-p sample to be matched (in the homology field) with analogous peptides and proteins found in the Asteraceae family. The selected peptides of a given amino acid sequence were included in the enzymatic (oxygenases, dehydrogenases, transferases) or plant immune proteins from the Asteraceae family. They are specified in Table 2. In the case of NWFKPG sequence fragment, there are suggestions of proteins from which they may originate. Additionally, for a 3038 *m*/*z* two equally likely sequence fragments were selected (FSRERD, NKFSRE). In the case of antimicrobial peptides, it was important to determine the pI value. In our mixture of 46 peptides from burdock, ten occurring in the largest amount were distinguished. Based on the data contained in Table 2, it can be concluded that the Br-p peptide mixture contained peptides with a pI in the range of 3.67–11.83. Among them there was a cationic peptide LRCDYGRFFASKSLYDPLKKRR with theoretically inhibitory activity on bacterial permease and with a pI of 10.18. Thus, due to the type of activity and cationic nature, the peptide may be responsible for antibiotic activity.

**Figure 3:**
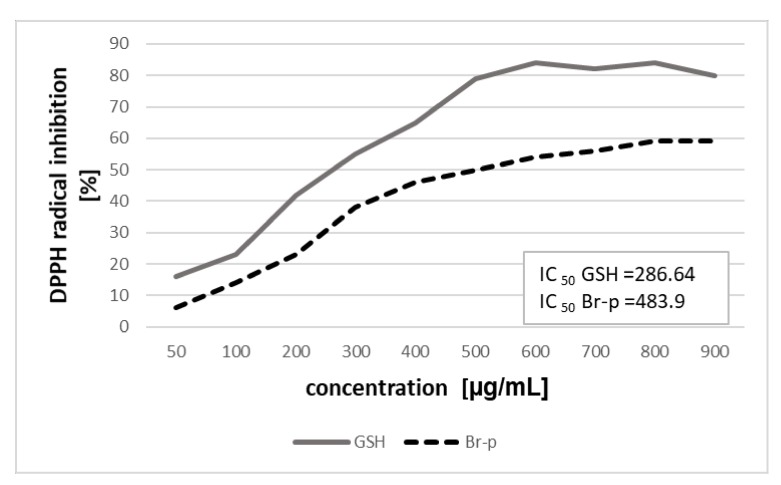
The 2,2-diphenyl-1-picrylhydrazyl radical scavenging activity of Br-p compared with standard GSH.

The next step was to determine the strength of scavenging the free radicals of Br-p in the DPPH assay. Such an activity of the peptide sample would be desirable when preparing an anti-acne formulation. In the experiment, radical scavenging assays were used to evaluate the activity of Br-p and glutathione (GSH) as a positive control (Figure 3). The DPPH free radical inhibition assay of the new peptide fraction Br-p revealed the potent antioxidant activity as observed through the plot of effective concentration (50–900 μg/mL) (Figure 3). The IC_50_ value of Br-p for DPPH inhibition was found to be 483.9 μg/mL. The results were comparable to that of the reference peptide GSH (tested at 50–900 μg/mL) and with IC_50_ of 286.64 μg/mL. In analyzing the sequence of peptides present in the sample, it can be seen that they contained fragments of peptides considered to be antioxidant, hence high antioxidant properties may result. Additionally, during the isolation process there is a possibility of nonspecific breaking of amino acid chains, resulting in the occurrence of small di- or tripeptides that are not observed on the MALDI spectrum. It may mean that there are many more peptide fragments with antioxidant activity in the Br-p sample.

**Figure 4:**
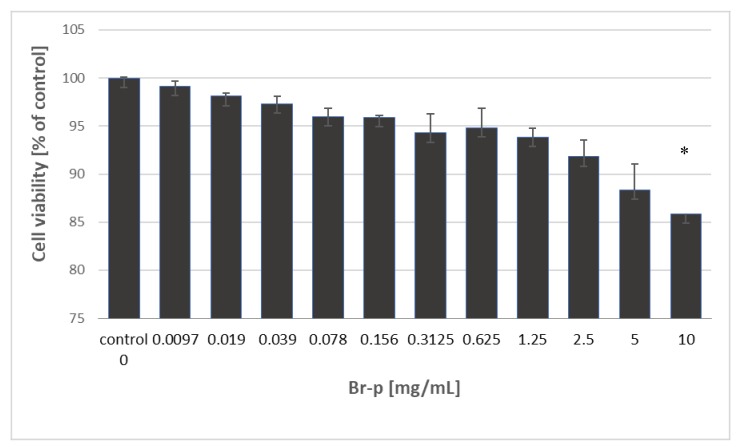
The graph of the human fibroblasts (BJ) viability depending on the concentration of Br-p against a control; * statistically significant results compared to the control.

The investigated Br-p peptides sample was evaluated for its cytotoxic activity towards the human skin fibroblast cell line (BJ) after 24 hours of incubation. Based on MTT assay results, the half maximal cytotoxic concentration (CC_50_) was determined and summarized in Figure 4.

Data show that Br-p peptides exhibited no cytotoxicity against BJ cells in the entire tested concentration range (0.0097–10 mg/mL). The fibroblast viability was maintained with a tested Br-p concentration range at a high level of over 85%. This result means that the CC_50_ values of Br-p peptides were higher than 10 mg/mL. Thus, for biocompatibility and selectivity determination of extracts, the CC_50_ value of Br-p was assigned as 10 mg/mL (Table 1). To estimate the in vitro therapeutic safety and efficacy of the Br-p peptide sample, the selectivity index (SI) was determined. SI index is defined as the ratio of cytotoxic activity (CC_50_) to antibacterial activity (MIC). High values of the selectivity index indicate that the tested sample may be more effective against bacterial strains and safer for Eukaryotic cells. Thus, SI values below 10 indicate lack of therapeutic safety, and SI values higher than 10 allow to perform in vivo evaluation [27]. It is extremely therapeutically important that the Br-p exhibited a favourable and highly safe therapeutic potential against acne strains *P. acnes* PCM 2334 and *P. acnes* PCM 2400, with safety SI values of 320 and 160, respectively. However, towards the tested Gram-negative bacteria (*E. coli* and *P. aeruginosa*) Br-p did not meet the safety requirements.

**Figure 5:**
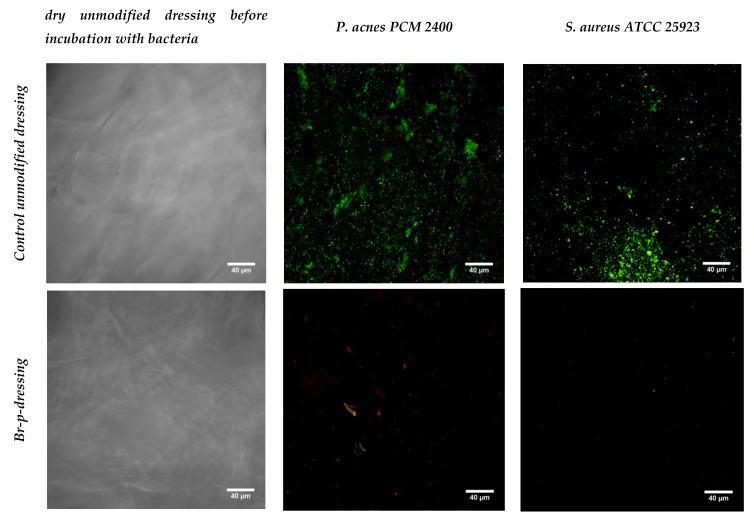
Confocal laser-scanning microscope (CLSM) images showing biofilm formation on unmodified and Br-p-modified dressings determined in a confocal microscope; magnification 400×; scale bar = 40 µm.

The last stage of research was the production of an exemplary functional anti-acne dressing modified with the Br-p peptide fraction from burdock root. The dressing material was created with genipine cross-linked chitosan and alginate. Obtained after freeze-drying, the hydrogel sheet composed of polysaccharides was a uniform, thin foam resistant to chipping or cracking. The control unmodified and modified with Br-p peptide dressings were immersed in a medium with a plankton form of acne strains. It was compared whether such planktonic forms of bacteria could create colonies resulting in biofilm formation to the same extent on control and modified materials. The conducted test imaged in a confocal laser-scanning microscope showed the beneficial effects of modification with Br-p (Figure 5). Namely, the confocal laser-scanning microscope (CLSM) images showed *P. acnes* or *S. aureus* biofilms on the surface of the dressing with viable and dead colonies (green and yellow-red fluorescence, respectively). The control dressings group demonstrated viable *P. acnes* or *S. aureus* bacteria, which formed green fluorescent biofilm architecture indicating that bacteria were viable during the evaluation period. Whereas, only red colonies of dead bacteria were seen on the tested dressings. Images show weaker or no adherence of tested bacteria to the Br-p-modified dressing in comparison to the unmodified control (Figure 5).

## 3. Discussion

Acne vulgaris is a chronic disease of the hair sebaceous gland accompanied by seborrhoea. It is not an infectious disease, but patients have a multiplication of *Propionibacterium acnes* [28]. Achermann’s team presented relative abundances of *Propionibacterium* species in different skin areas, proving that these bacteria are not only present in the face area, resulting in not only cosmetic trouble, but make it difficult to carry out surgical procedures in a larger area [28].

The roots of burdock (*Arctium lappa L.*) are commonly used as herbal medicine based on its valuable phytochemical content. Pereira et al. [29], Gentil et al. [30] and Pirvu et al. [31] showed antibacterial activity against Gram-positive and Gram-negative bacteria of crude extracts from burdock, and Knott et al. and Jingvi et al. [32,33] proved skin improvement when using burdock extracts. One of the molecules in burdock roots responsible for antibacterial properties can be peptides because every organism (from microorganisms to Mammalia) has an innate defense system, which is determined by the presence of, among others, antimicrobial peptides (AMPs) [34]. Therefore, it was desirable in our work to purify the crude extract of burdock root to assess the biological activity of the peptide solution. The presence of 46 fractions of peptides with molecular masses lower than 5000 in Da Br-p was determined by MALDI TOF. The 10 major peaks were subjected to BioTools RapidDeNovo sequencing (Figure 2). The determined theoretical pI values confirmed the presence of some cationic peptides with a preference for antibacterial activity with pI values of 11.83, 10.18, 9.49, and 8.15. Based on Table 2, we can theoretically assume that the peptide LRCDYGRFFASKSLYDPLKKRR with pI of 10.18 and KK amino acid fragment, with activity indicated as a bacterial permease ligand, may be responsible for the antibiotic activity. In the mechanism of energy transduction in bacterial membranes, a bulk-phase, transmembrane electrochemical ion gradient is an important problem and concerns, e.g., secondary active transport, oxidative phosphorylation, and rotation of the bacterial flagellar motor. Transport involves substrate-specific membrane proteins that catalyze equilibration or uphill translocation of solute across a membrane. These carrier proteins are named transporters, cotransporters, symporters, or permeases. Thus, permeases are involved in active transport of energy sources, e.g., glucose, fructose, various sugars, and sugar alcohols. Thus, permeases, by providing the bacterial cell with a source of energy, may be the target of molecular inhibition of bacterial growth [35].

Both burdock root samples (Br-f, Br-p) obtained in subsequent purification stages were evaluated for antimicrobial character. The data of bacterial growth inhibition show that burdock root polypeptide samples had the ability to inhibit the growth of acne bacteria (Figure 1). The results indicate that the antibacterial nature of the samples increased with the number of purification steps, as the Br-p-purified sample was at least twice as strong as Br-f. The MIC value confirmed that Br-p had stronger antibacterial properties than the mixture of crude Br-f polypeptides (Table 1). Br-p possessed bactericidal nature against all Gram-positive strains, whereas Br-f was only active against anaerobic Gram-positive strains. The tested preparations from burdock roots did not inhibit the activity of Gram-negative strains.

The narrow spectrum demonstrated by burdock root samples, limited only to Gram-positive bacteria, may be beneficial. It suggests that chemotherapy with a single drug may be used in the fight against acne, which is preferred for the identified pathogen, minimizes the impact on the body’s physiological flora, and reduces side effects and toxicity. Our tests also showed that a Br-p sample in an acid environment pH (BHI broth and BHI agar pH 6.0) had antibacterial activity. This is a promising result and therapeutically useful for cosmetic formulations requiring acidic pH. Besides, antibacterial peptides active under acidic conditions have a protective effect against bacterial infections (the skin, oral cavity, urinary tract, and vagina possess acidic pH). A Korean team [36] noted that the high antibacterial properties of Nod1 and Nod2 peptides isolated from *Nordotis discus* in an acidic environment might be useful when these peptides are applied as skin therapy in pH of about 5.5.

The clinically important measurement results of the selectivity index SI value are interesting. The high values of the selectivity index indicated that the tested sample may be more effective against bacterial strains and safer for Eukaryotic cells. Thus, SI values below 10 determine a lack of therapeutic safety, and SI values higher than 10 allow to perform in vivo evaluation [27]. SI values of Br-p for Gram-positive strains were at least 20 and, even compared to acne strains, had an extremely favorable selectivity of up to 360. Low toxicity and good antibacterial activity result in a safe value on therapeutic index, suggesting that the obtained purified burdock root peptides could be promising as an anti-acne agent. Other authors also report favorable, high SI values of tested antimicrobial peptides (AMPs) from plants and the possibilities of their application in the cosmetics, medical, food, or agriculture industries [37,38,39,40,41].

On the other hand, Kumar et al. [42] showed that, although many AMPs have reached clinical trials, not many of them have been approved by the US Food and Drug Administration (FDA) due to issues with toxicity. Authors have pointed out strategies to improve the activity and biocompatibility of AMPs, such as chemical modifications and the use of delivery systems [41]. Another Georgian-American team of scientists working on de novo AMP synthesis pointed out that the design of AMPs with high therapeutic indexes, low cost of synthesis, high resistance to proteases, and high bioavailability remains a challenge. Authors have developed a digital tool to predict AMP algorithms for the design of de novo AMPs, and short peptides with high therapeutic indexes against Gram-negative bacteria have been designed [43].

Due to the nature of acne skin diseases, anti-acne drugs should also have an antioxidative nature. Free radicals are not only dangerous for normal and acne skin, but they also can lead to a variety of various human diseases (cardiovascular diseases, rheumatoid arthritis, Alzheimer’s disease, and cancer). According to some of the authors, regardless of the solvent used, the crude burdock root extract possessed significant antioxidant activity, as determined with DPPH; thus, crude extract seems to be a promising antioxidant [23,26,44,45,46].

In our work the DPPH free radical scavenging assay was performed to assess the antioxidant capacity of the Br-p peptide samples against the free DPPH radical. The experiment showed potent antioxidant activity (range 50–900 μg/mL) (Figure 3). The IC_50_ value of Br-p for DPPH inhibition was found to be 483.9 μg/mL. The possibility of scavenging free radicals through burdock peptides is promising when applied to dermatological cosmetic formulations.

It is significant that also proteins, polypeptides and peptides are proper antioxidants because they can inhibit biomolecule oxidation though some pathways (including inactivation of reactive oxygen species, scavenging free radicals, chelation of pro-oxidative transition metals, and reduction of hydroperoxides). Changes to the tertiary protein structure increase the accessibility of amino acid residues that can scavenge free radicals and chelate pro-oxidative metals. Peptides, not proteins, show the most promise as proteinaceous antioxidants, and a number of studies have shown that they have a substantially higher activity than proteins with a whole, intact structure [47,48]. For diseases with keratinolytic dysfunction, it is beneficial to combat both free radicals and the bacteria that cause these diseases, with a lack of cytotoxicity. Low-weight molecular fractions of burdock root peptides exhibit such combined characteristics.

In the last stage of the experiments, we made an antibacterial and antioxidant active dressing for acne skin. The dressing base created de novo with polysaccharides (chitosan and sodium alginate) was crosslinked by genipin and modified with Br-p peptides. The hydrogel thus obtained, after freeze-drying, had a dry foam structure that was tested for bacterial biofilm adhesion. The Br-p-modified dressing was found to have beneficial antibacterial properties; it showed no adhesion or poor adhesion of the tested bacteria to the surface compared to the unmodified control material, as evidenced by CLSM images (Figure 5).

To use the developed modification of active Br-p peptide from burdock roots to create commercial dressings, in the future, research should be carried out with clinical bacterial strains isolated from dermatological patients. It would be advisable to carry out in vivo tests that would confirm or exclude the effectiveness of antibacterial dressings in the prevention and treatment of peri-operative acne skin infections.

## 4. Materials and Methods

### 4.1. Extraction and Fractionation of Antibacterial Peptides

Burdock roots (*Arctium lappa L*, *Bardanae radix* dried, Kawon, Gostyń, Poland) (100 g) were grated in a mortar, and proteins were extracted with buffer (10 mM Na_2_HPO_4_, 15 mM NaH_2_PO_4_, 100 mM KCl, pepsin 1.5 mg/g defatted powder, 2 mM EDTA, pH 6.7) by sonication at 37 °C in a series of 3 × 15 min. Ammonium sulphate was added to get 30% saturation, and precipitate obtained after 1h at room temperature was removed after centrifugation (15 min at 4000× *g*). Collected supernatant was adjusted to 80% ammonium sulphate saturation, and precipitated proteins (after centrifugation at 4000× *g* for 10 min at 4 °C) were resuspended in distilled water and dialyzed against distilled water using 2000 Da cut-off dialysis tubing (Sigma-Aldrich, Saint Louis, MO, USA). The peptide fraction after dialyse was next purified by size exclusion chromatography on a Sephadex G-50 (Sigma-Aldrich) on a column (72 × 2 cm) using the same buffer as that used in extraction. Each eluted fraction (4 mL) was collected and monitored for peptides at λ = 230 nm. Additionally, fractions were collected and analyzed using a screening antibacterial test on solid medium (Br-f fractions). The samples active against bacteria were combined together and ultrafiltrated through a 10 kDa cut-off membrane (Amicon). The antibacterial activity was again controlled after freeze-drying and treated as an active peptide fraction from burdock roots (Br-p fractions). The freeze-dried extract Br-p sample was weighed, the overall process yield was calculated, and then 20 mg/mL stock solutions were prepared.

### 4.2. MALDI Molecular Mass Weight Determination

MALDI technique was used to monitor the peptide purification. For measuring molecular masses of the peptides, the Br-p sample was analyzed on a matrix-assisted laser desorption/ionization time of flight mass spectrometer Ultraflextreme (MALDI TOF/TOF; Bruker, Bremen, Germany). MALDI analysis was performed according to a previously proposed procedure [49]. Briefly, 0.5 µL of peptide sample was mixed with the same volume of matrix saturated solution containing α-cyano-4-hydroxycinnamic acid (HCCA, Bruker, Bremen, Germany) and 2,5-dihydroxy benzoic acid (DHB, Bruker, Bremen, Germany). Next, the obtained mixture was spotted on an AnchorChip MALDI plate (Bruker, Bremen, Germany) with prespotted HCCA in acetone. Analyzed Br-p peptides were subjected to mild ionization using MALDI-TOF in the linear mode and recorded in active positive reflector mode within the 800–5000 m/z range with laser frequency 500 Hz and 2000 shot counts. The collected spectrum was smoothed using the Savitzky–Golay method, baseline corrected (Top Hat baseline algorithm), and the list of peaks for the signal-to-noise ratio of >3 was generated using flex Analysis 3.0 software (Bruker, Bremen, Germany). The major peaks with signal-to-noise ratios greater than 20 were assigned to RapidDeNovo sequencing using fragmentation spectra obtained in MS/MS tandem mode. In this aim, the MS/MS data were loaded and recognized automatically, b- and y- ions were detected, and the software predicted the sequence or its fragment based on the peaks which were presented. Automatically predicted sequences were given with their probability of occurrence. The most probable sequence (or sequence fragment) was next analyzed by the BLAST algorithm for finding regions of amino acid similarity in order to assign them to proteins from which they originated. We selected "protein-protein BLAST" as a search algorithm which simply compares a peptide fragment to a protein database. We chose only proteins from Asteraceae family to compare and designated peptide fragments were 100% identical to the matched proteins. Knowing a sequence of a given protein enabled the determination of the peptide sequence with a given mass (*m*/*z*) using PeptideMass ExPASy [50]. The peptide sequence was then analyzed for active fragments in the BIOPEP-UWM database, while theoretical biological activity and isoelectric point were indicated [51].

### 4.3. Antimicrobial Assays

Antibacterial assays were done using the micro-aerobic strains *Propionibacterium acnes* PCM 2400 and *Propionibacterium acnes* PCM 2334 as well as the aerobic strains *Staphylococcus aureus* ATCC 25923, *Staphylococcus epidermidis* ATCC 12228, *Pseudomonas aeruginosa* ATCC 27853, and *Escherichia coli* ATCC 25992 as a microbial cause of acne appearance. Before setting up the experiments, micro-aerobic bacteria were grown separately on BHI agar (BioMaxima S.A., Lublin, Poland), pH 6.0, under anaerobic conditions for 48 h at 37 °C or aerobic bacteria in M-H agar (BioMaxima S.A. Poland), for 24 h at 37 °C. Bacterial strains were diluted in broth (BHI broth (BioMaxima S.A. Poland) in the case of micro-aerobic bacteria, or M-H broth (BioMaxima S.A. Poland) in the case of aerobic bacteria) to get 1.5 *×* 10^8^ CFU/mL of bacteria culture for microbiologic assays.

### 4.4. Antibacterial Activity in Solid Medium

The preliminary antibacterial activity of the burdock roots samples against pathogenic Gram-positive micro-aerobic and aerobic bacterial acne was evaluated by measuring the zones of inhibition in the disk diffusion method [52]. Antibacterial disc diffusion assays were carried out on Petri plates with solid medium (BHI agar (pH 6.0 [28], or M-H agar). Appropriate strain cultures were separately spread over the agar surface with a sterile cotton swab. Next, each sample (10 μL) was placed on Petri plates with an agar medium. After 24 h of incubation at 37 °C (for aerobic strains) or 48 h at 37 °C (for micro-aerobic strains), zones of microbial growth created around the tested burdock samples were measured and recorded as the diameters of inhibition (mm).

### 4.5. Broth Microdilution Method

A broth microdilution method was used to evaluate the minimum inhibitory concentration (MIC) according to the CLSI document (CLSI performance standards for antimicrobial susceptibility testing, Eighteenth International Supplement, CLSI document M7-MIC, Clinical Laboratory Standards Institute, Wayne) with some modifications. The lowest concentration of the tested compound (μg/mL) which did not result in any visible growth of bacteria was considered as the MIC value. A serial doubling dilution of the burdock samples was prepared in 96-well plates (200 μL per well). A suitable medium (M-H broth, BHI broth) was used as a diluent. The final concentrations of burdock samples were 4000–7.812 μg/mL. Finally, 2 μL of inoculum of the tested bacterial strain (1.5 *×* 10^8^ CFU/mL) was added to each well. The tests were performed either at 37 °C for 24 h (aerobic strains) or at 48 h (micro-aerobic strains). After incubation, the panel was digitally analyzed at 600 nm using the microplate reader Bio Tech Synergy (USA) with a dedicated software system. The growth intensity in each well was compared to the negative (clear broth) and positive (broth with inoculum) controls. Additionally, minimal bactericidal concentration (MBC) was obtained by spreading on agar 5 µL of medium from the clear well, which did not show any visible growth after incubation during the MIC test. The plates were incubated at 37 °C for 24 h, and the MBC was defined as the lowest concentration of sample without bacterial growth. Each experiment was repeated in triplicate.

### 4.6. Cytotoxicity Evaluation

The cell culture experiment was performed using a human skin fibroblast cell line (BJ cells from American Type Culture Collection ATCC, UK). BJ cells were cultured in EMEM supplemented with 10% FBS, 100 U/mL penicillin, and 100 μg/mL streptomycin and maintained at 37 °C in a humidified atmosphere of 5% CO_2_ and 95% air. The cells were seeded in 96-well plates in 100 μL of a complete growth medium (supplemented with 10% FBS) at a concentration of 1.5 *×* 10^4^ cells/well and incubated for 24 h at 37 °C in a humidified atmosphere of 5% CO_2_. After this time, the Br-p sample was firstly diluted in DMSO to obtain the stock solution at 10 mg/mL, and the solution was further diluted in culture medium supplemented with 2% FBS. Subsequently, the culture medium was replaced with 100 μL of the serial dilutions of the Br-p peptide sample. Untreated cells were used as a control of cytotoxicity, and different concentrations of DMSO were used as a solvent control. The cell cultures were incubated at 37 °C for 24 h. Cytotoxic effects were estimated using the MTT test. The experiment was repeated in three separate measurements. The half-maximal cytotoxic concentration (CC_50_) was defined as the sample concentration required to reduce cell viability to 50%. The CC_50_ values were calculated via four-parameter nonlinear regression analysis (GraphPad Prism 5, version 5.04) and were presented as mean values ± standard deviation (SD).

### 4.7. Antioxidant Activity—DPPH^●^ Method

The present study evaluated the antioxidant activity of Br-p samples by employing a diphenylpicrylhydrazyl (DPPH^●^) (Sigma-Aldrich, USA) radical scavenging assay based on the Abdel Moneim method [53]. This assay was conducted spectrophotometrically in 96-wells plates using digital reader Bio Tech Synergy (USA). Color disappearance of DPPH (100 μL 0.1 mM in 95% ethanol) after 20 min was monitored at 517 nm. This DPPH decolouration, or lack thereof, was caused by the presence of 10 μL tested samples in the concentration range 50–800 µg/mL. Due to the precipitate formed (in some samples) during incubation (as a result of the ethanol of DPPH with the sample combination), after 20 min of incubation the mixture had to be centrifuged, the sediment rejected, and the clear solution read in a clean 96-well plate. Ascorbic acid and glutathione (GSH) were used as standards for the 2,2-diphenyl-1-picrylhydrazyl scavenging assay. The following equation was used to calculate the percentage of DPPH decolorization of the sample:

% DPPH^●^ radical inhibition = [(Abs control − Abs sample)/Abs control] *×* 100 (1)

where Abs is absorbance at 517 nm.

Radical scavenging activity was expressed as the inhibition concentration (IC_50_), i.e., concentration of sample required to inhibit 50% of DPPH free radicals. IC_50_ was determined graphically from the curve plot between the percentages of DPPH scavenging activity and the sample’s concentration.

### 4.8. Anti-Acne Dressing Creation

Dressing material for acne skin disease treatment was created using natural polysaccharides crosslinked with genipin (Sigma-Aldrich, USA). Briefly, 1.25% chitosan (75–85% deacetylation degree, 50–190 kDa, Sigma-Aldrich, USA) and 1.25% sodium alginate (Sigma-Aldrich, USA) were separately dissolved in 10 mL of 0.5% acetic acid and water, respectively. Then, alginate was poured in portions into the chitosan standing on a magnetic stirrer. After the two polysaccharides were completely mixed, calcium β-glycerophosphate (10 mg/mL) used as inorganic filler was added and mixed for 30 min at 37 °C. The obtained hydrogel was divided into two parts: one as an unmodified control and another modified with Br-p (0.05 mg/mL) as the active substance. Finally, 1% genipin (0.6 µg/mL) was added as the bifunctional cross-linker, with further mixing until a homogeneous mixture was obtained. The resulting modified and unmodified gels were transferred quantitatively into appropriate forms and left for 6 h at room temperature for crosslinking. The final stage of dressing formation was deep frozen (−70 °C) and freeze-dried (LYO GT2 Basic SRK SystemTechnik, GmbH, Germany), resulting in discs with a diameter of about 6.5 mm and thickness of about 0.2 mm. Modified and unmodified dressings were sterilized using a plastic/paper peel pouch in ethylene oxide prior to further testing.

### 4.9. Dressing Seeding with Bacterial Strains for Biofilm Formation Determination

Dry dressing samples (unmodified dressing as a control; dressing modified by Br-p) were washed in 100 µL of PBS and then transferred to the bottoms of 24-well polystyrene plates (CytoOne, USA). Each well with a disc sample inside was filled up with 1000 µL of appropriate broth (BHI or M-H). Finally, 10 µL of inoculum (1.5 *×* 10^8^ CFU/mL) was added to each well. For mono-species biofilm assay it was *P. acnes* PCM 2400 and *S. aureus* ATCC 25923. Sterility controls (only M-H or BHI broth) were included in all experiments. To allow biofilm formation, i.e., the adhesion of planktonic forms of bacteria in the colonies attached to the biomaterial, the plates were incubated twice as long. Namely, plates with aerobic bacteria were incubated for 48 h, and with micro-aerobic bacteria 96 h, both at 37 °C. The tests were performed using three replicates.

### 4.10. Confocal Laser-Scanning Microscopy Biofilm Visualization

The viability of bacteria and their adhesion to the material surface were determined using double fluorescent staining for both live and dead bacteria using Viability/Cytotoxicity Assay kit for Bacteria LIVE/DEAD Cells (Biotium, Hayward, CA, USA). Dressing samples with bacterial suspensions were prepared for confocal microscopy assay after the incubation. First, the medium from wells was removed and gently washed twice with 200 µL of 0.9% NaCl, to remove the loosely adherent planktonic bacteria and to leave only the biofilm attached to the material. Next, samples were transferred into fresh wells and filled with 200 µL of 0.9% NaCl and LIVE/DEAD dye. The solution of this dye was prepared by mixing 1 µL of DMAO with 1 µL of EthD-III in 8 µL of 0.9% NaCl. Three microliters of such obtained live/dead dye solution was added to disc-containing wells with 200 µL of PBS. The samples were incubated for 15 min at room temperature in the darkness, and bacterial colonies attached to dressing samples were visualized using a confocal microscope (CLSM) with dedicated software.

### 4.11. Statistical Analysis

The results are expressed as mean ± RSD. Statistical analysis was performed by analysis of variance (ANOVA) tests, and statistical significance was set accordingly at the P = 0.05 level.

## 5. Conclusions

Our research allowed us to obtain promising results regarding the antibacterial and antioxidant activities of the tested burdock peptides. The obtained solution containing 46 Br-p peptides with a value below 5000 Da from burdock roots had anti-acne properties and were not toxic to fibroblast cultures. These properties of active peptides have allowed them to be used to produce therapeutic dressing material (Br-p/chitosan/sodium alginate) for use in infections against Gram-positive bacteria in acne skin disease. One of the peptides is LRCDYGRFFASKSLDPLKKRR, whose theoretical pI and bacterial permease ligand activity may possess antibiotic, anti-oxidative and anti-seborrheic properties. However, in-depth research is necessary on the biochemical analysis of the isolated Br-p peptide fraction.

## Figures and Tables

**Table 1:** Antibacterial activity (MIC, MBC/MIC ratio) and cytotoxicity (CC_50_ as fibroblast activity and selectivity index SI) caused by burdock root samples.

Sample	*S. aureus* ATCC 25923				*S. epidermidis* ATCC 12228				*P. acnes* PCM 2334				*P. acnes* PCM 2400				*E. coli* ATCC 25992				*P. aeruginosa* ATCC			
	MIC	MBC MIC	CC_50_	SI	MIC	MBC MIC	CC_50_	SI	MIC	MBC MIC	CC_50_	SI	MIC	MBC MIC	CC_50_	SI	MIC	MBC MIC	CC_50_	SI	MIC	MBC MIC	CC_50_	SI
**Br-f**	500	8	>10	>20	500	8	>10	>20	250	4	>10	>40	500	4	>10	>20	>2000	-	>10	-	>2000	-	>10	-
**Br-p**	250	4	>10	>40	250	4	>10	>40	31.25	2	>10	>320	62.5	2	>10	>160	>2000	-	>10	-	>2000	-	>10	-

Br-f: burdock root samples after initial separation, Br-p: burdock root final peptide sample. MIC (µg/mL); MBC/MIC ratio, CC _50_ (mg/mL), SI.

**Table 2:** Peptide Br-p sequence fragments assigned in RapidDeNovo sequencing; theoretical activity character and pI.

Sequence Fragment Identify by Rapid de Novo	*m*/*z*	S/N *	Asteraceae Family Proteins Possible Source of Peptides **	Probable Peptide Sequence	Activities ***	Theoretical pI ****
VQGR	2470	24.2	photosystem I P700 chlorophyll a apoprotein A1 (*Arctium lappa*)	QPRALSIVQGRAVGVTHYLLGGIA	Antioxidant: **YLL** Activating ubiquitin-mediated proteolysis: **RA**	**10.84**
WHM	2696	388.2	ribulose-1.5-bisphosphate carboxylase/oxygenase large subunit. partial (*Arctium eriophorum*)	FTQDWVSLPGVLGHPWGNAWHMPA	Antioxidant: **PWG, AW, WG**	5.97
GAAV	2700	1180.0	ribulose 1-5 bisphosphate carboxylase oxygenase. partial (*Arctium lappa*)	QPGVPPEEAGAAVAAESSTGTWTTVWT	Antioxidant: **TW, VW, GAA, GTW**	3.67
WFANH	2714	73.4	histone-binding protein RBBP4 (*Artemisia annua*)	YDWFANHNLLWPSLSCRWGPLE	Antioxidant: **RW, LW**	5.32
TLAW	2718	55.9	photosystem I P700 chlorophyll a apoprotein A2 (*Arctium lappa*)	WRGYWQELIETLAWAHERTPLA	Antioxidant: **AH, EL, AW**	5.50
RFFASKS	2720	35.1	fructan:fructan 1-fructosyltransferase (*Arctium lappa*)	LRCDYGRFFASKSLYDPLKKRR	Immunomodulating: **YG** Antioxidant: **LY, LK** Bacterial permease ligand: **KK**	**10.18**
STAG	2737	36.4	NADH dehydrogenase subunit F. partial (*Arctium lappa*)	SWLYSPIFAIIAWSTAGLTAFYMC	Antioxidative **LY, AW**	5.24
EYPTGR			Acyl-CoA dehydrogenase. conserved site-containing protein (*Cynara cardunculus var. scolymus*)	IQCLGGNGYM NEYPTGRYLR DAKL	Antioxidative: **QCL** Alpha-glucosidase inhibitor: **YP**	**8.15**
WEYPT			transposase. MuDR. MULE transposase domain protein (*Artemisia annua*)	AEAVNFVGEYHEWEYPTHIKPII	Antioxidative: **KP** alpha-glucosidase inhibitor: **YP, EA**	4.87
PFFY	2783	27.2	NADH dehydrogenase subunit 7 (*Arctium lappa*)	LWLGPFMADIGAQTPFFYIFRER	Antioxidative: **LW**	6.07
NWFKPG	3034	163.9	Resistance protein candidate. partial (*Lactuca sativa*)	TLLVLDDVDHIDQLEALAGDLNWFKPG	Antioxidative: **KP** Alpha-glucosidase inhibitor: **EA** Activating ubiquitin-mediated proteolysis: **LA**	3.90
			nematode resistance-like protein. partial (*Artemisia tridentata*)	DVNHKDQLEALAGNCNWFKP GSRIIIT	Antioxidative: **KD, KP, NHK** Alpha-glucosidase inhibitor: **EA** Activating ubiquitin-mediated proteolysis: **LA**	6.75
			TMV resistance protein N-like (*Helianthus annuus*)	DDVDHIDQLEALAGEPNWFKPGSRVII	Activating ubiquitin-mediated proteolysis: **LA**	4.23
FSRERD	3038	52.8	cyclic dof factor 1-like (*Lactuca sativa*)	SITSSNSRNEEFSRERDSES GVFTPKT	Hypolipidemic: **EF** CaMPDE inhibitor: **EF**	5.00
			Leucine-rich repeat-containing protein (*Artemisia annua*)	VIGVSVGLVFIGGVMFLRHWFSRERDA	Immunostimulating: **GVM** Antioxidative: **RHW**	**9.49**
NKFSRE			Pollen receptor-like kinase 4 (*Helianthus annuus*)	FKRLRRLRSIFLTANKFSREIPTDA	CaMPDE inhibitor: **KF**	**11.83**

* Signal-to-noise ratio on mass spectrum. ** Protein sequences matched to sequence in databases in Basic Local Alignment Search Tool (BLAST). *** Activities determined in BIOPEP-UWM database of proteins and BIOPEP-UWM database of bioactive peptides. **** Theoretical pI determined in *PeptideMass* ExPASy.

## Supplementary Materials

The following are available online at https://www.mdpi.com/1420-3049/25/9/2027/s1, Figure S1: title: Br-p sample analyse in MALDI/TOF. MS/MS fragmentation spectra for RapidDeNovo sequencing.

## Abbreviations

MIC minimum inhibitory concentration; MBC minimum bactericidal concentration; SI selectivity index; EDTA ethylenediaminetetraacetic acid; MALDI TOF/TOF matrix-assisted laser desorption/ionization time of flight mass spectrometer; SD standard deviation; ATTC American Type Culture Collection; PCM Polish Collection of Microorganism; BHI Brain Heart Infusion; M-H Mueller–Hinton; CFU colony-forming units; PBS phosphate-buffered saline; DMSO dimethyl sulfoxide; EMEM Eagle’s minimum essential medium; FBS fetal bovine serum; MTT methylthiazolyldiphenyl-tetrazolium bromide; CLSM confocal laser-scanning microscope; GSH glutathione; pI isoelectric point.
