# Supplementary figures and images for: Anti-Acne Action of Peptides Isolated from Burdock Root—Preliminary Studies and Pilot Testing
Source: Molecules. 2020 Apr 27;25(9):2027. doi: 10.3390/molecules25092027 (PMC7248785; doi:10.3390/molecules25092027)

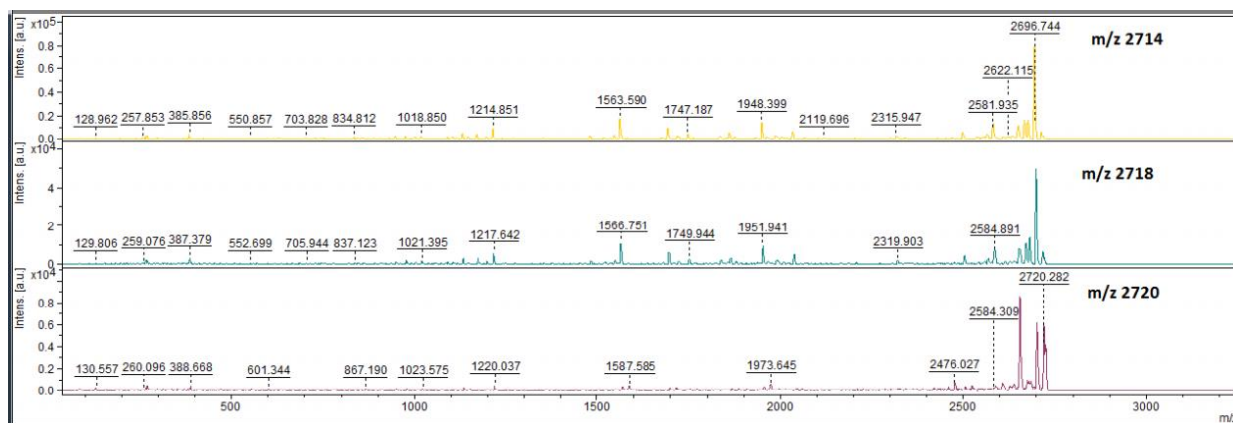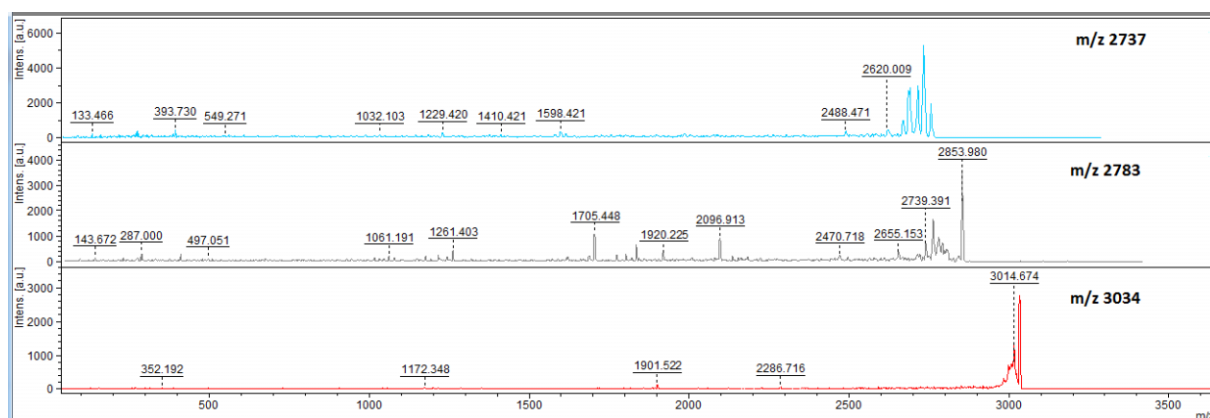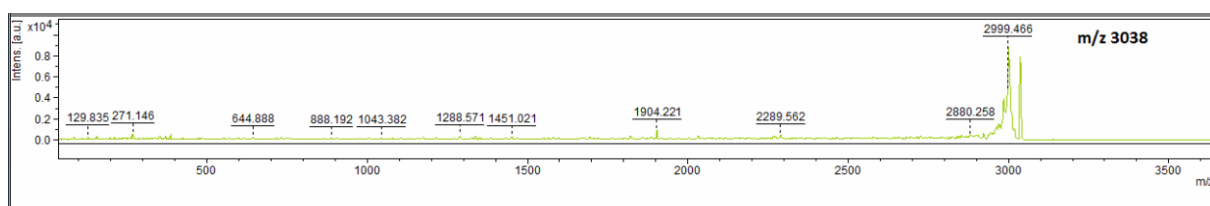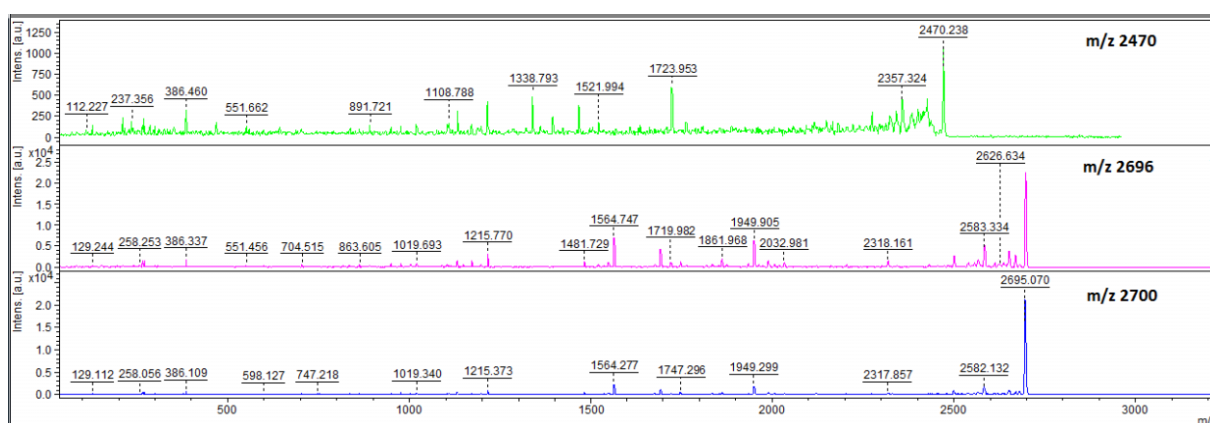

Supplement: Supplementary file 1 [file molecules-25-02027-s001.pdf]
